# Poststroke Oropharyngeal Dysphagia: A Clinical Approach to Diagnosis and Management

**DOI:** 10.14309/ajg.0000000000004029

**Published:** 2026-04-22

**Authors:** Edoardo Vincenzo Savarino, Jose Maria Remes-Troche, Adriana Lazarescu

**Affiliations:** 1 Gastroenterology Unit, Department of Surgery, Oncology and Gastroenterology, University of Padua, Padua, Italy;; 2 Medical Biological Research Institute, Universidad Veracruzana, Veracruz, Mexico;; 3 Hospital Español de Veracruz, Veracruz, Mexico;; 4 Department of Medicine, Faculty of Medicine and Dentistry, Division of Gastroenterology, University of Alberta, Edmonton, Alberta, Canada.

## EPIDEMIOLOGY

Oropharyngeal dysphagia (OD) is a prevalent yet often underrecognized disorder characterized by impairments in the oral and/or pharyngeal phases of swallowing (1). Symptoms include choking, coughing, nasal regurgitation, and pharyngeal residue, leading to aspiration pneumonia, malnutrition, dehydration, and impaired quality of life. OD arises from neurological disorders, structural abnormalities, pharmacological effects, autoimmune diseases, and age-related changes and is particularly prevalent in older adults (1). Prevalence varies across care settings: approximately 36.5% in hospitals, 42.5% in rehabilitation facilities, 50.2% in nursing homes, and up to 82.4% in individuals older than 80 years (2). Stroke is the most frequent neurological cause of OD. Poststroke dysphagia (PSD) affects 29%–81% of patients with acute stroke, and 11%–50% remain symptomatic at 6 months (3). PSD compromises swallowing safety and efficacy, increases aspiration pneumonia risk, and adversely impacts nutritional status and psychological well-being (3). Spontaneous recovery of swallowing occurs in many patients during the first-week poststroke, driven by cortical reorganization of the bilateral swallowing motor network; however, persistent dysphagia beyond 3 months is associated with impaired cortical activation and predicts worse long-term outcomes. These findings highlight the need for early diagnosis and a multidisciplinary management strategy. We present a clinical vignette that synthesizes current evidence.

## CLINICAL PICTURE

A 74-year-old man was admitted with fever, productive cough, and respiratory distress. His family reported progressive confusion and poor oral intake after a right middle cerebral artery ischemic stroke 3 weeks prior. Medical history included hypertension, atrial fibrillation (on apixaban), type 2 diabetes mellitus, and hyperlipidemia. Examination revealed lethargy, a wet gurgly voice, drooling, and coarse crackles with decreased breath sounds in the right lower lung field, together with left-sided hemiparesis and hyperreflexia consistent with his prior stroke. Laboratory findings included white blood cell 15,000/µL, C reactive protein 80 mg/L, and mild hypoxemia (PaO_2_ 72 mm Hg on 2 L O_2_). Chest radiograph confirmed right lower lobe pneumonia. Initial management included empiric IV piperacillin-tazobactam, supplemental oxygen, and nil per os status pending swallowing evaluation by a speech-language pathologist (SLP). This case illustrates the consequences of delayed dysphagia assessment after stroke.

## DIAGNOSIS

Patients with OD may present with coughing or choking on swallowing, nasal regurgitation, repeated forceful swallowing, or immediate bolus holdup. Alternatively, silent aspiration may lead to aspiration pneumonia, as illustrated above. The 2019 American Heart Association/American Stroke Association Stroke guidelines strongly recommend dysphagia screening before any oral intake in all patients with stroke, with formal assessment within 24 hours for those who fail initial screening (4). Appropriate tools include the Volume-Viscosity Swallow Test (5) and the Toronto Bedside Swallowing Screening Test (6). The stroke subtype influences the pattern of dysphagia: Anterior circulation strokes tend to impair oral phase function; posterior circulation and brainstem strokes more severely affect pharyngeal triggering and laryngeal function; hemorrhagic strokes generally carry higher dysphagia risk than ischemic strokes of similar size. In addition, bilateral hemisphere involvement and comorbid cognitive impairment are associated with particularly poor prognosis for swallowing recovery and should prompt early multidisciplinary intervention.

Patients failing screening require instrumental assessment. Videofluoroscopy (VFS) and fiberoptic endoscopic evaluation of swallowing (FEES) are complementary examinations (7). VFS uses fluoroscopy with contrast medium to evaluate all phases of swallowing, detect silent aspiration, test compensatory strategies, and assess upper esophageal sphincter (UES) opening; it cannot assess UES relaxation and requires transport to the radiology suite (7). FEES consists of transnasal endoscopy to visualize the pharynx and larynx during swallowing, detect silent aspiration and secretion accumulation, and assess laryngeal sensitivity without radiation exposure; it is ideal for bedside assessment in acute stroke (8). High-resolution pharyngoesophageal manometry, with or without impedance, quantifies pharyngeal and UES pressure dynamics; distinguishes insufficient UES relaxation from reduced hyolaryngeal excursion; and is reserved as a second-line test when VFS and/or FEES are inconclusive (9). Key characteristics of these tests are summarized in Table 1.

**Table 1.**
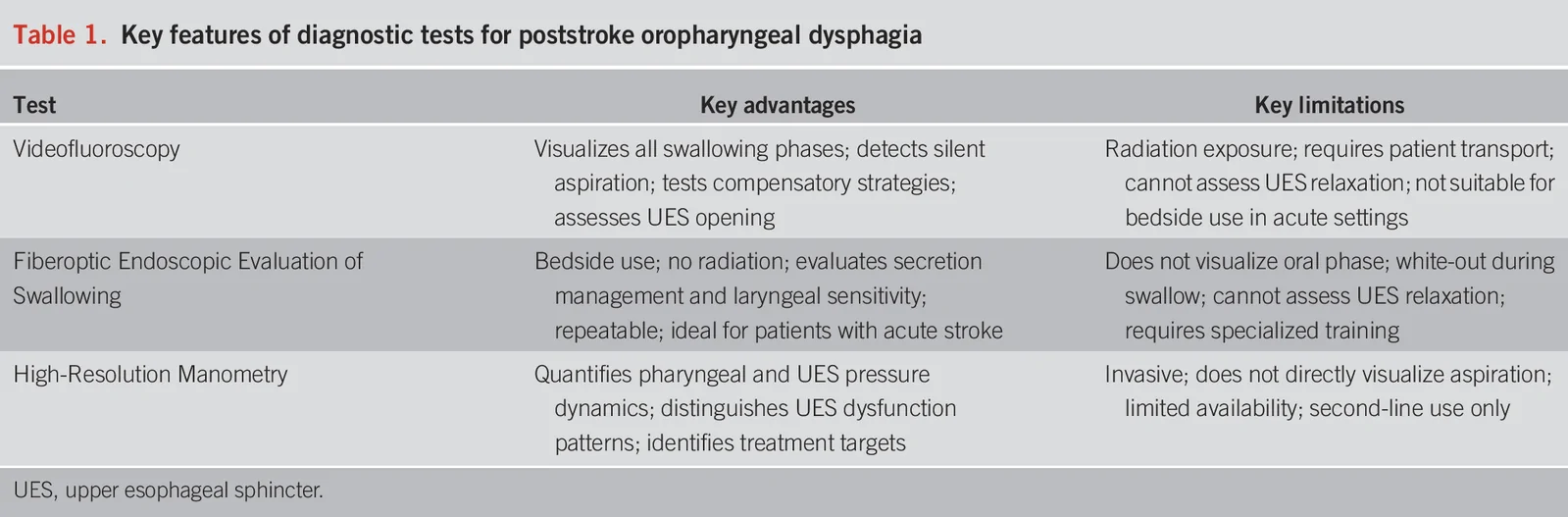
Key features of diagnostic tests for poststroke oropharyngeal dysphagia

## CLINICAL PICTURE CONTINUED

The SLP documented poor oral control, delayed swallow initiation, and frequent coughing with thin liquids. VFS confirmed silent aspiration of thin liquids, pharyngeal residue, and UES dysfunction. FEES confirmed secretion pooling in the pyriform sinuses and vallecula and silent thin-liquid aspiration without cough response. Given the high aspiration risk, a percutaneous endoscopic gastrostomy (PEG) tube was considered for long-term enteral feeding. Neurology and rehabilitation services were engaged for comprehensive poststroke care. Ideally, dysphagia screening should have been initiated within 24 hours of stroke admission: Early identification would have allowed timely dietary modification and swallowing rehabilitation, potentially preventing aspiration pneumonia and its associated morbidity.

## MANAGEMENT

**Figure 1.**
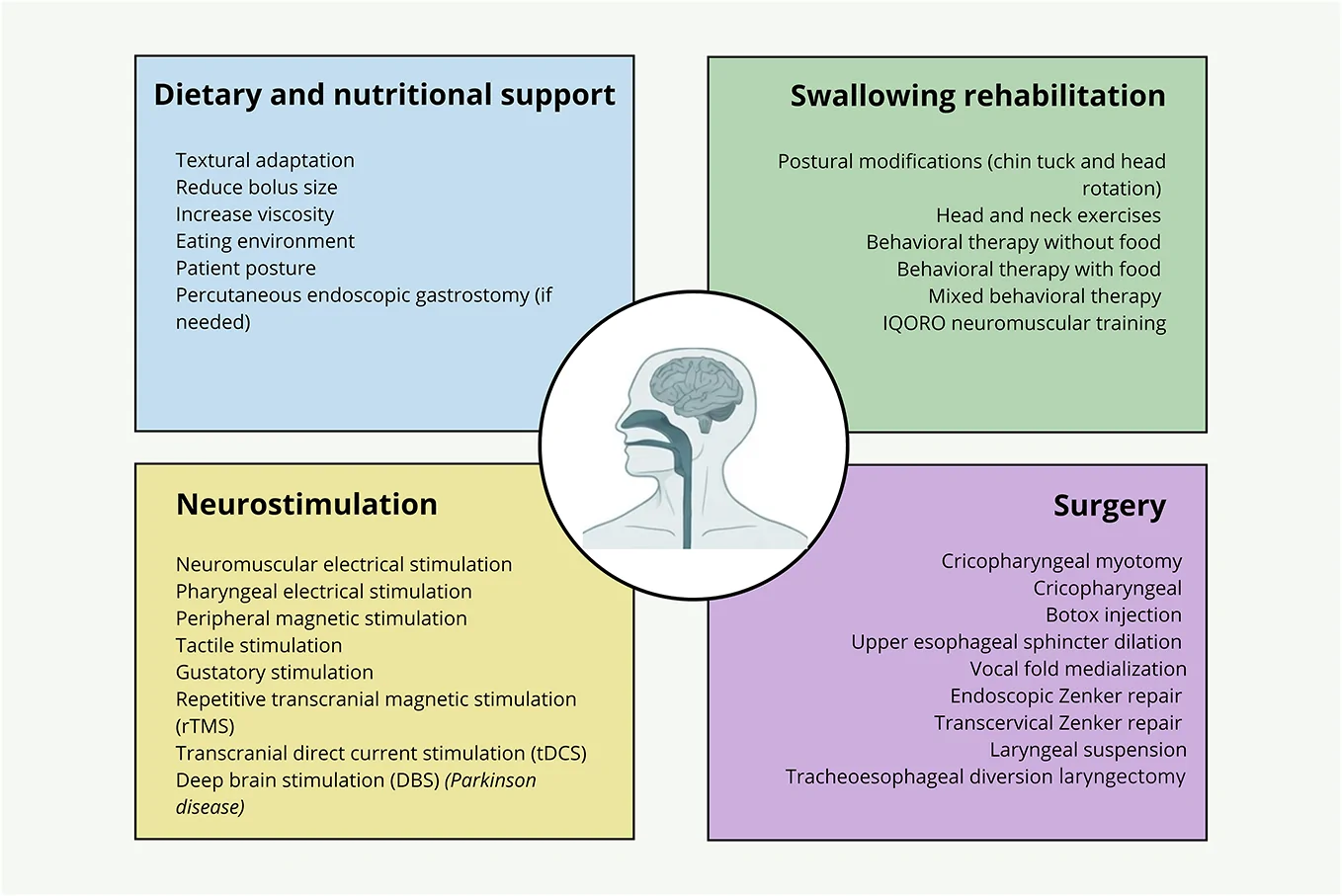
Therapeutic approaches for oropharyngeal dysphagia, organized by domain.

**Figure 2.**
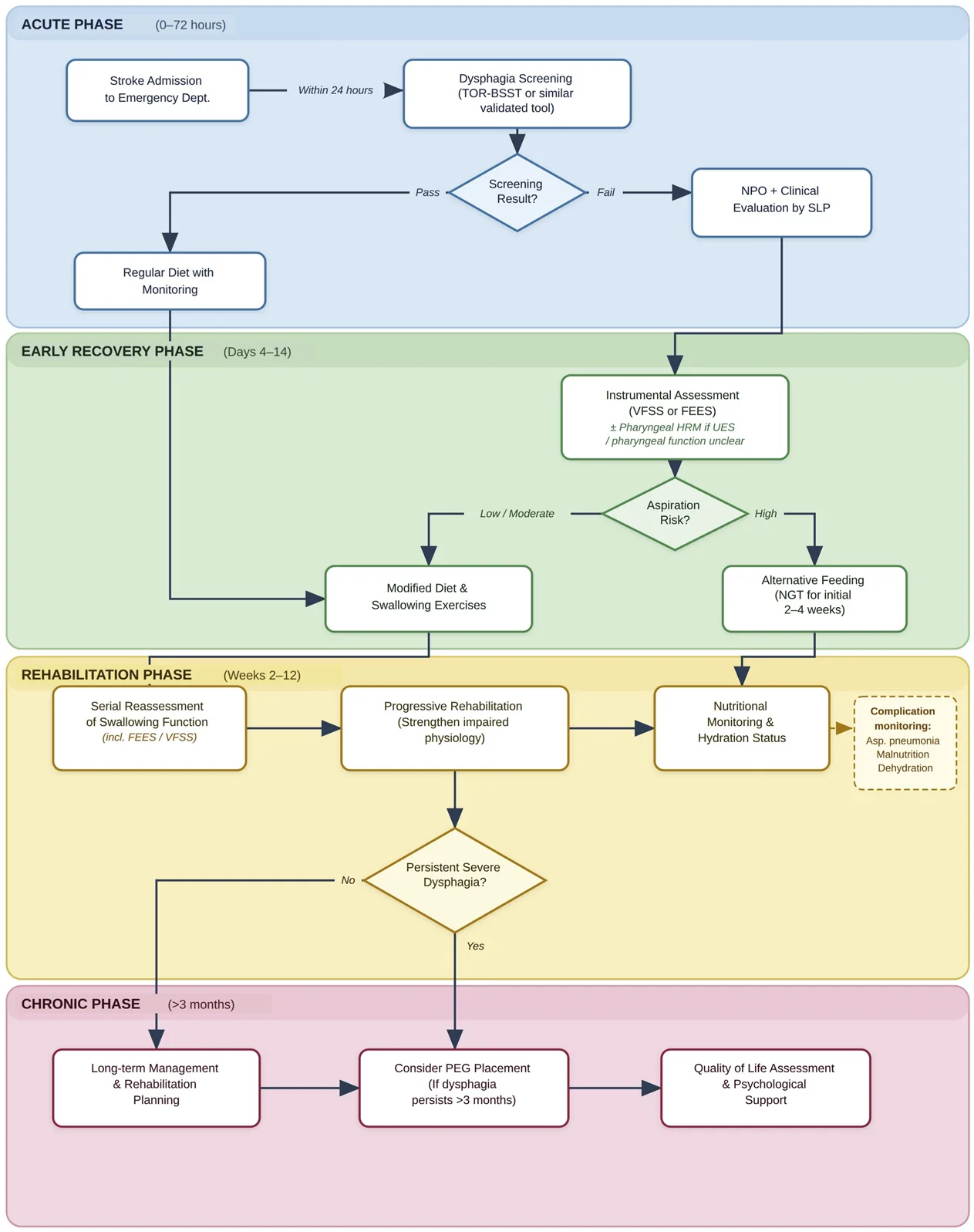
Algorithm for the management of poststroke oropharyngeal dysphagia across 4 phases of recovery. FEES, flexible endoscopic evaluation of swallowing; NGT, nasogastric tube; NPO, nil per os (nothing by mouth); PEG, percutaneous endoscopic gastrostomy; SLP, speech-language pathologist; TOR-BSST, Toronto Bedside Swallowing Screening Test; VFSS, videofluoroscopic swallowing study.

The primary goals in managing OD are to ensure adequate nutrition and hydration, improve quality of life, and prevent aspiration pneumonia. A multidisciplinary team including SLPs, dietitians, specialized nurses, occupational therapists, and rehabilitation specialists is central to optimal outcomes. Therapeutic approaches for PSD, illustrated in Figure 1, include dietary and nutritional support, swallowing rehabilitation, neurostimulation, and surgical interventions. A proposed management algorithm for PSD is shown in Figure 2.

### Dietary and nutritional support

Texture-modified diets and thickened liquids, classified using the IDDSI framework, reduce aspiration risk and improve swallowing safety (10). For patients unable to maintain adequate oral intake, enteral feeding is recommended. Nasogastric feeding is preferred initially; PEG is appropriate for dysphagia persisting beyond 8 weeks. The FOOD trial demonstrated that early PEG placement within 7 days was associated with poorer functional outcomes compared with nasogastric feeding, supporting a deferred approach to gastrostomy (11). The European Society for Clinical Nutrition and Metabolism guidelines endorse enteral feeding in severe dysphagia, although it may not fully prevent aspiration pneumonia (12).

### Compensatory maneuvers and swallowing rehabilitation

Postural modifications such as chin tuck and head rotation reduce aspiration risk. Rehabilitation exercises—including the Shaker head-lifting exercise to enhance UES opening (13) and behavioral therapies with or without food (14)—aim to restore swallowing strength, coordination, and sensory feedback through neuroplasticity. Recovery is generally more favorable after anterior circulation strokes than after brainstem lesions, which should guide rehabilitation planning. A clinically important distinction exists between compensatory strategies—which improve immediate swallowing safety without altering underlying physiology—and rehabilitative techniques targeting neuroplasticity-driven recovery; both are integral to a comprehensive PSD management programme.

### Neurostimulation

Peripheral approaches (neuromuscular electrical stimulation, tactile, and gustatory stimulation) and central approaches (repetitive transcranial magnetic stimulation, transcranial direct current stimulation) aim to modulate the neural networks governing swallowing (15). Among peripheral approaches, pharyngeal electrical stimulation—delivered through transnasal catheter to the pharyngeal mucosa—has accumulated the most consistent randomized controlled trial evidence in PSD, modulating cortical excitability of the unaffected hemisphere to promote swallowing recovery. Broader clinical adoption of neurostimulation therapies requires confirmation from adequately powered multicenter trials.

### Surgical management

Surgical interventions are reserved for cases refractory to conservative treatment. UES dysfunction may be addressed with cricopharyngeal myotomy, botulinum toxin injection, or mechanical dilation. Zenker diverticulum is managed endoscopically or by open transcervical repair with myotomy. Aspiration prevention procedures include laryngeal suspension, tracheoesophageal diversion, and partial laryngectomy (15). Outcomes depend on careful patient selection and multidisciplinary coordination.

## CONCLUSION

Timely recognition and management of OD are crucial to prevent aspiration pneumonia, malnutrition, and dehydration. Raising awareness in elderly and neurologically vulnerable populations is essential for early diagnosis and intervention. A multidisciplinary approach integrating SLPs, dietitians, nurses, and rehabilitation therapists optimizes patient outcomes. In the poststroke setting, management should be guided by stroke type and location, evidence-based screening protocols, and individualized rehabilitation planning. Emerging neurostimulation therapies, particularly pharyngeal electrical stimulation, offer additional promise for patients with persistent deficits refractory to conventional rehabilitation.

## CONFLICTS OF INTEREST

**Guarantor of the article:** Edoardo Vincenzo Savarino, MD, PhD.

**Specific author contributions:** E.V.S., J.M.R.-T., and A.L.: contributed equally to design, data collection, writing, and final approval.

**Financial support:** None to report.

**Potential competing interests:** Edoardo Vincenzo Savarino has served as a speaker for Abbvie, Abivax, Agave, AGPharma, Alfasigma, Apoteca, Biosline, CaDiGroup, Celltrion, Dr. Falk, EG Stada Group, Fenix Pharma, Galapagos, Johnson&Johnson, JB Pharmaceuticals, Innovamedica/Adacyte, Eli Lilly, Malesci, Mayoly Biohealth, Montefarco, Novartis, Omega Pharma, Pfizer, Rafa, Reckitt Benckiser, Sandoz, Sanofi/Regeneron, SILA, Sofar, Takeda, Tillots, and Unifarco; has served as a consultant for Abbvie, Agave, Alfasigma, Biogen, Bristol-Myers Squibb, Celltrion, Dr. Falk, Eli Lilly, Fenix Pharma, Ferring, Giuliani, Grunenthal, Johnson&Johnson, JB Pharmaceuticals, Merck & Co, Nestlè, Pfizer, Reckitt Benckiser, Sanofi/Regeneron, SILA, Sofar, Takeda, and Unifarco; he received research support from Bonollo, Difass, Pfizer, Reckitt Benckiser, Sanofi/Regeneron, SILA, Sofar, Unifarco, and Zeta Farmaceutici. J.M. Remes-Troche is an adviser and advisory council member for Asofarma, Carnot, PRO.MED.CS Praha a.s., and Pisa. He is a speaker for Asofarma, Abbot, Carnot, Chinoin, Ferrer, Johnson and Johnson, Medix, and Medtronic. AL: none.
